# A Novel Low-Density-Biomass-Carbon Composite Coated with Carpet-like and Dandelion-Shaped Rare-Earth-Doped Cobalt Ferrite for Enhanced Microwave Absorption

**DOI:** 10.3390/molecules29112620

**Published:** 2024-06-02

**Authors:** Tao Shang, Hongwei Zhu, Yichun Shang, Ruixia Wu, Xuebing Zhao

**Affiliations:** 1 Department of Physics Science and Technology, Baotou Teacher’s College, Baotou 014030, China; 2 Key Laboratory of Industrial Biocatalysis, Ministry of Education, Tsinghua University, Beijing 100084, China; 3 Institute of Applied Chemistry, Department of Chemical Engineering, Tsinghua University, Beijing 100084, China; 4 College of Science, Inner Mongolia University of Technology, Hohhot 010051, China

**Keywords:** microwave absorption, biomass, porous carbon, rare-earth doping, ferrite, composite materials, special morphology

## Abstract

A novel low-density composite for the absorption of microwaves was prepared by loading La-doped spinel cobalt ferrite (La-CFO) onto biomass carbon (BC) derived from corn stalks using a hydrothermal method. This composite (La-CFO@BC) not only maintained the advantageous properties of low density and abundant porosity, but also exhibited a unique morphology, with La-CFO displaying a carpet-like structure interspersed with dandelion-shaped particles. The incorporation of La-CFO effectively tuned the electromagnetic parameters of the composite, thereby improving its impedance-matching attributes and its ability to absorb microwave radiation. At a frequency of 12.8 GHz for electromagnetic waves and with a thickness of 2.5 mm, La-CFO@BC demonstrated remarkable performance in microwave absorption, attaining a noteworthy minimum reflection (*R*_Lmin_) of −53.2 dB and an effective absorption bandwidth (EAB) of 6.4 GHz. Furthermore, by varying the thickness of the La-CFO@BC within the range of 1.0 to 5.5 mm, the EAB could be broadened to 13.8 GHz, covering the entire X-band, the entire Ku-band, and a substantial portion of the C-band. This study demonstrated that La-CFO@BC was a promising alternative for electromagnetic wave attenuation, which offered superior performance in microwave absorption.

## 1. Introduction

Amidst the rapid evolution of contemporary communication technologies, especially following the rollout of the 5G network, electronic devices have become increasingly prevalent in enhancing human living standards. However, the abundant electromagnetic radiation has intensified the electromagnetic pollution in the environment, which not only provokes electromagnetic interference issues but also poses severe threats to telecommunications security, national defense security, and human health [1,2,3]. Given the complexity of these issues, the progress in the design and synthesis of electromagnetic wave (EM) absorbers has emerged as a pivotal area of research. Specifically, these materials must have features such as thinness, lightweight, broad bandwidth coverage, and high absorption capacity.

In recent years, due to their inherent lightweight nature and specific absorption capabilities, these carbon-based materials have gained increasing attention from researchers. Biomass carbon (BC) has unique pore structures and suitable electrical conductivity, and it is environmentally friendly, low cost, and easily available [4,5,6]. Additionally, the residual N, S, and Si elements in BC can create self-doping effects, making it a potential absorbing material. However, in the absorption of EW by BC the contribution of magnetic loss is relatively minor, and the main contribution arises from dielectric loss, thereby limiting its absorption capacity. Previous research has shown that loading magnetic particles onto the surface of carbon materials is an effective means to enhance magnetic loss in carbon materials [7,8,9,10]. Wang et al. [11] successfully prepared porous jute biochar (PJBC) composed of Fe_3_O_4_ nanoparticles using a chemical coprecipitation method. Research has shown that the minimum *R*_L_ value of Fe_3_O_4_/PJBC composite material is −39.5 dB at a frequency of 6.4 GHz. Wang and co-workers [12] prepared a nano porous carbon (PC)@NiFe_2_O magnetic composite material using pomelo peels as raw material. The composite material demonstrated remarkable microwave-absorption capabilities, achieving the *R*_Lmin_ of −50.8 dB precisely at a frequency of 13.4 GHz. Furthermore, with a thickness of 2.5 mm, it exhibited an absorption bandwidth spanning 4.9 GHz. Liu et al. [13] conducted a significant study where they synthesized Fe_3_O_4_/CF (Fe_3_O_4_/carbon fiber) composite material. In this synthesis, bagasse waste was utilized as the primary raw material. The composite material exhibited a maximum *R*_L_ value of −48.2 dB at 15.6 GHz, simultaneously achieving a wide EAB of 5.1 GHz at a thickness of 1.9 mm. Among various magnetic substances, cobalt ferrite (CoFe_2_O_4_) stands out for its stable chemical and excellent magnetic properties [14,15]. Additionally, it has been confirmed that the magnetic loss of iron oxide can be improved by doping it with rare-earth ions [15,16,17]. The slight addition of rare-earth ions modifies the coercivity, magnetic crystal anisotropy field, and diffusion activation energy of ferrite crystals, leading to an increase in magnetic-hysteresis loss. Rare-earth doping also boosts the natural resonance absorption peak and domain wall resonance absorption peak of ferrite, alters peak positions, and broadens absorption peaks, thereby enabling regulation of the material’s ability to absorb EW.

However, investigations into the composite of rare-earth-doped magnetic particles with BC and their synergistic effect on the material’s absorption performance have been rarely reported. Therefore, in this work, we have selected corn stalks, one of the most abundant agricultural waste biomass resources in China, as the raw material to use to synthesize BC. Subsequently, we employed a hydrothermal method to load the rare-earth-La-doped magnetic cobalt ferrite, CoLa_0.12_Fe_1.88_O_4_ (La-CFO), onto the BC, resulting in a novel composite microwave absorber, La-CFO@BC. The advantages of this composite material lay in the complementary interactions between cobalt ferrite doped with rare-earth elements and biomass-derived carbon, as well as with the unique carpet-like morphology exhibited by La-CFO, which together enhanced the impedance-matching conditions and attenuation capacity of the composite material. Consequently, La-CFO@BC exhibited exceptional EW attenuation characteristics. Notably, at the La-CFO@BC thickness of 2.5 mm, the composite achieved an *R*_Lmin_ of −53.2 dB for electromagnetic waves, accompanied by an EAB of 6.4 GHz. These remarkable properties rendered the La-CFO@BC composite a promising candidate for microwave-absorption applications.

## 2. Results and Discussion

### 2.1. Configuration Analysis

**Figure 1:**
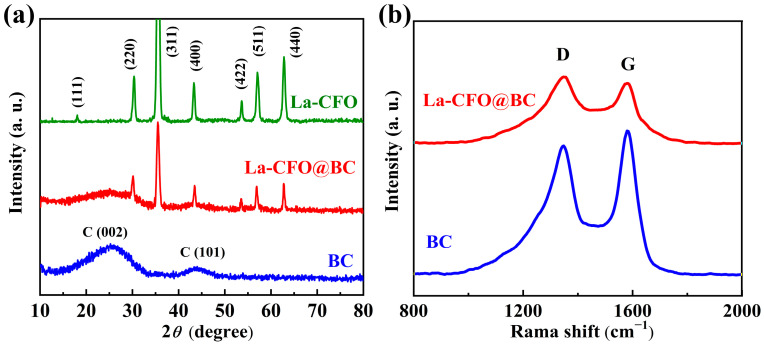
The XRD and the Raman spectroscopic signatures of La-CFO@BC composite. (**a**) X-ray diffraction patterns; and (**b**) Raman spectroscopic signatures.

Using the PANalytical Empyrean for X-ray diffraction analysis (XRD), the crystalline configurations of BC, La-CFO@BC, and La-CFO composites were examined. From Figure 1a, it can be observed that biomass-char BC exhibited two broad diffraction peaks near 2*θ* of 25.2° and 44°. These observed reflections in the X-ray diffraction patterns matched the crystallographic orientations of the graphite’s (002) and (101) planes. The wider hump shape indicated that the corn stalk BC obtained in the experiment has a very low graphitization degree, and that the char was mainly present in the form of amorphous carbon. Additionally, seven distinct diffraction peaks were visible for La-CFO and La-CFO@BC samples, which corresponded to the (511), (440), (422), (400), (311), (220), and (111) crystal planes of the spinel ferrite face-centered cubic structure. These results indicated the CoLa_0.12_Fe_1.88_O_4_ and the La-CFO@BC contained structures present in the form of a spinel ferrite face-centered cubic crystal structure with good crystallinity. Furthermore, in the XRD pattern of La-CFO@BC, the absence of any distinctive peaks associated with La compounds suggested that La^3+^ had been successfully incorporated into the lattice of CoFe_2_O_4_.

Figure 1b displays the Raman spectroscopic signatures of BC and La-CFO@BC composites. In both samples, distinct peaks were evident at 1348 cm^−1^ (corresponding to the D-band) and 1584 cm^−1^ (associated with the G-band). The D-band is attributed to the vibrational modes stemming from the appearance of amorphous sp³ carbon bonds of disordered sp^3^ carbon configurations and structural defects within the graphitic. Alternatively, the G-band signifies the vibrational frequencies stemming from the stretching of sp^2^ carbon configurations in the ordered lattice framework. Consequently, ID/IG, which is the ratio of the intensities of two peaks, is a widely used indicator of the degree of graphitization and structural defects in the material [18,19]. Upon analysis, it has been determined that BC exhibited ID/IG values of 0.93, whereas the corresponding values for La-CFO@BC were found to be 1.05. The higher ID/IG ratio of La-CFO@BC indicated lower graphitization due to the increased defects and disrupted carbon-atom ordering during the activation process and during La-CFO loading. The introduced defects within the material can function as potent centers for electrode polarization within electromagnetic fields, thereby augmenting the electric dipole moments and enhancing the dielectric losses of the material towards electromagnetic waves [20,21]. Additionally, a pinning effect can be exerted on the magnetic moment reversal by these defects, making the reversal process more challenging, which consequently leads to an increase in magnetic hysteresis losses [22]. Furthermore, the presence of defects significantly impacts the conductivity of materials, as previously reported [23,24]. Specifically, defects within BC materials have the potential to increase the resistance of their inherent conductive networks, thereby enhancing the thermal effects associated with current flow and ultimately leading to increased conductivity losses. Collectively, these attributes contribute towards the enhanced electromagnetic wave-absorption capabilities of the La-CFO@BC.

**Figure 2:**
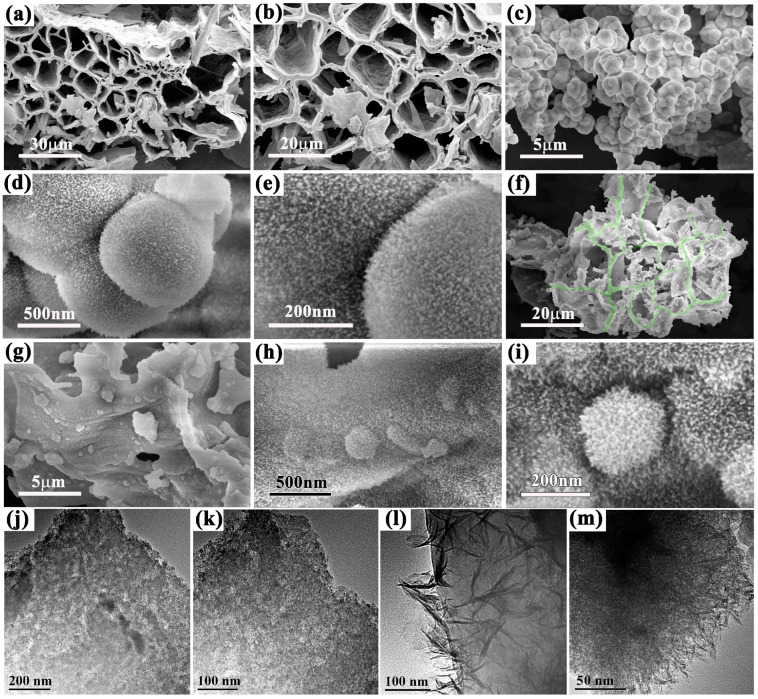
The images from SEM of the BC (**a**,**b**) La-CFO (**c**–**e**) and La-CFO@BC (**f**–**i**) particles, and the TEM images of the BC (**j**,**k**) and La-CFO (**l**,**m**) particles.

The microscopic structures of BC, La-CFO and the composite La-CFO@BC were examined using the SEM as well as the TEM, as illustrated in Figure 2. According to Figure 2a,b, BC retained the 3D-network-porous-cell-wall structure with abundant pore-size distribution, mainly ranging from micro to sub-micro scales. The pore walls covered a wide range of thicknesses and, importantly, they contained numerous thin-layer curled and corrugated distributions. This special structure can enhance the specific surface area of the material, thereby providing a larger interface for potential applications. Additionally, the porous structure promoted the development of an interconnected three-dimensional (3D) conductive network. Meanwhile, it provided expanded regions for electromagnetic wave reflection and scattering. These characteristics were beneficial for improving the material’s microwave-absorption performance at different wavelengths. Figure 2b–d presents SEM images of La-CFO at different magnifications. It can be seen that La-CFO accumulated and was distributed in a granular manner, with particle sizes ranging from 700 nm to 1 μm. Additionally, the particle’s surface was covered with fine fluffy protrusions, giving the entire particle a dandelion-like morphology. As depicted in Figure 2e,f, the La-CFO@BC maintained a 3D-network-porous structure (the pore walls were rendered in green in the picture), and the surface of BC was densely coated with carpet-like, fluffy La-CFO interspersed with embedded dandelion-shaped La-CFO particles. Notably, the size of these embedded La-CFO particles was smaller compared to those in the pure La-CFO samples, with a distribution primarily ranging from 200 nm to 500 nm.

Figure 2j,k are the TEM images of BC, which clearly showed an abundance of microporous and mesoporous structures within its structure. Figure 2l,m are the TEM images of La-CFO@BC. It can be observed that there are also abundant microporous and mesoporous pore structures in La-CFO@BC. Among them, due to the relatively large thickness of the composite material, the pore structure was not obvious in the TEM image labeled (l). In addition, it can be observed that the surface of BC was covered with carpet-like La-CFO fluffy protrusions, and that the surface of dandelion-shaped La-CFO particles also exhibited abundant fluffy protrusions. The distinctive morphology of the material significantly contributed to increasing its specific surface area, which strengthened the surface polarization and improved the absorption of EW. Furthermore, this unique morphology played a pivotal role in modulating the surface impedance of BC, adding to its overall absorption functionality. The complete coverage of La-CFO on the BC surface effectively guided the electromagnetic waves towards the material’s interior, thus improving the internal absorption. This dual-faceted approach could significantly boost the material’s overall electromagnetic wave-absorption capabilities as discussed in the later sections.

**Figure 3:**
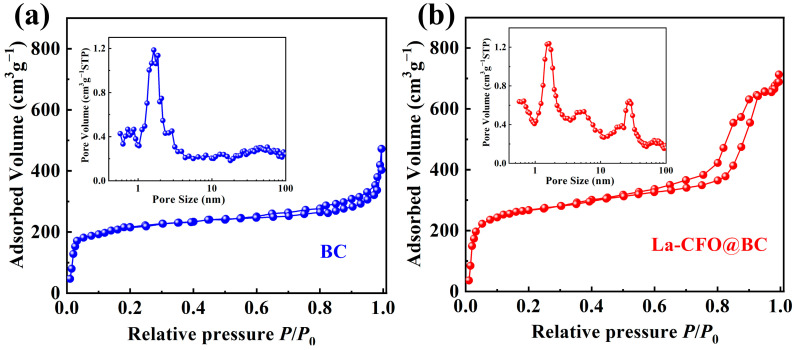
N_2_ isotherm adsorption/desorption curve and pore-size distribution curve of BC (**a**) and La-CFO@BC (**b**).

To investigate the detailed surface area and porosity profiles of BC and La-CFO@BC, we conducted measurements using nitrogen (N₂) adsorption/desorption isotherms. As depicted in Figure 3a,b, both BC and La-CFO@BC exhibited combined features of Type II and Type IV isotherms, indicative of a coexistence of microporous and mesoporous architectures. Compared to BC, the adsorption capacity of La-CFO@BC was significantly increased. This was due to the presence of carpet-like fluffy and dandelion-shaped La-CFO, which strengthened the surface-adsorption capability of La-CFO@BC. Additionally, the gaps between BC and La-CFO further contributed to the enhanced pore-adsorption capacity of La-CFO@BC. Notably, La-CFO@BC exhibits a more pronounced hysteresis loop compared to BC. This observation could be attributed to the formation of capillary condensation between the micropores in BC and the gaps between BC and La-CFO, making desorption more difficult.

The pore-size distribution curve presented in Figure 3a,b was derived from the analysis of the adsorption data within the respective isotherms. It can be observed that both BC and La-CFO@BC had hierarchical pore structures composed of micropores, mesopores, and macropores at the microscopic level. Specifically, BC was primarily characterized by micropores, which were mainly distributed around 2 nm. La-CFO@BC was dominated by a combination of micropores and mesopores, exhibiting primary distributions centered at 2 nm and 28 nm, respectively. Overall, there was a significant increase in the total pore volume of La-CFO@BC compared to BC. Using the BET method, the specific surface areas of BC were found to be 753.5 m^2^/g, whereas for La-CFO@BC it was 989.6 m^2^/g. Such large specific surface areas were beneficial for enhancing the material’s electrode polarization and multiple reflections of EW, ultimately contributing to improved microwave-absorption properties of the materials.

**Figure 4:**
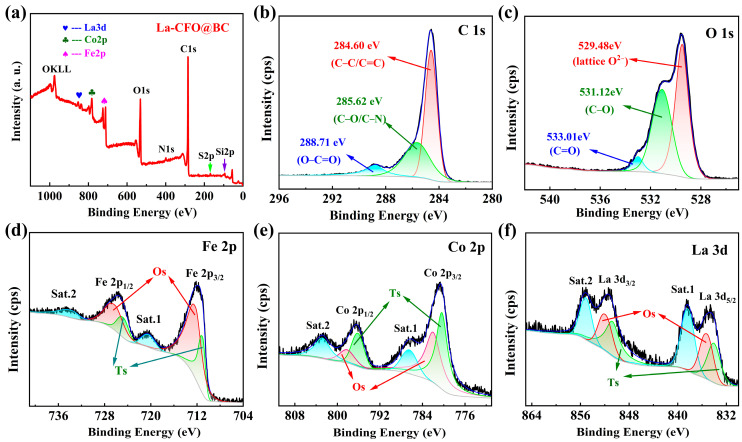
XPS survey spectra of La-CFO@BC. (**a**) The full spectrum of XPS; (**b**) C 1s; (**c**) O 1s; (**d**) Fe 2p; (**e**) Co 2p; and (**f**) La 3d.

The types, chemical states, and electronic structures of surface elements in the sample were studied using XPS spectroscopy. Figure 4a shows the survey XPS spectrum of La-CFO@BC, where eight elements, including C, O, N, S, Si, Co, Fe, and La could be identified. Figure 4b,c displays the C 1s and O 1s XPS spectra of La-CFO@BC. By using the Lorenz–Gaussian curve-fitting method, the C 1s spectral peak can be decomposed into three distinct sub-peaks, corresponding to the O-C=O, C-O/C-N, and C–C/C=C chemical bonds of the carbon element in BC, which are positioned at 288.71 eV, 285.62 eV, and 284.60 eV [19,21,25]. Similarly, the O 1s spectrum peak could be fitted into three sub-peaks: the one at 529.48 eV corresponding to the lattice O^2−^ ions in La-CFO, and the other two sub-peaks at 533.01 eV and 531.12 eV were identified as representing the C=O and C-O bonds originated from carbonized residual oxygen elements in BC. Furthermore, it can be deduced that the residual elements of N, S, and Si observed in the spectra were remnants after the carbonization of BC. These residual elements in BC had high polarity and tended to form more polar functional groups [26,27,28]. The presence of polar functional groups has been favorably documented as enhancing the absorption proficiency of the material [29]. Consequently, BC may have good absorption properties. Furthermore, the presence of these residual elements has the potential to increase the surface activity of biomass carbon by generating additional surface-active groups and elevating its surface energy [30,31]. This increase subsequently facilitates the interaction of the biomass carbon with electromagnetic waves, thereby improving its wave-absorption capabilities.

Figure 4d is the detailed XPS spectrum of La-CFO@BC for Fe 2p. Due to spin-orbit splitting, two dominant peaks were observed at 725.70 eV and 711.74 eV. The spectral features align with the spin-orbit split components of Fe 2p_1/2_, and Fe 2p_3/2_, respectively. Additionally, the observation of secondary peaks situated at elevated binding energies, approximately 6 eV above the primary peaks, indicates the existence of Fe^3+^ ions in their oxidized state, as reported in references [32,33]. Furthermore, the Fe2p peak exhibits an asymmetric shape, indicating that Fe exists in multiple chemical states within the compound. By decomposing the Fe 2p_1/2_ as well as Fe 2p_3/2_ peaks, two sub-peaks were obtained, corresponding to Fe^3+^ ions occupying octahedral interstitial sites (Os) and tetrahedral interstitial sites (Ts) in spinel cobalt ferrite. Table 1 provides the binding-energy positions of the Fe^3+^ sub-peaks. To ascertain the proportional percentage composition of Fe^3+^ ions across the two distinct states, we analyzed the integral area ratio of the corresponding sub-peaks, yielding values of 71.96% for the Os state and 28.04% for the Ts state, respectively.

Figure 4e displays the detailed XPS spectrum of La-CFO@BC for Co 2p. Two dominant peaks were observed. Due to spin-orbit splitting, the one at 796.43 eV was attributed to the Co 2p_1/2_ spin-orbit component, whereas another one at 780.60 eV corresponds to the Co 2p_3/2_ spin-orbit component. The presence of strong high-energy side peaks at 802.10 eV and 785.75 eV indicates the oxidation state of Co as Co^2+^ [34,35]. By decomposing the Co 2p_1/2_ and Co 2p_3/2_ peaks, two sub-peaks were obtained, corresponding to Co^2+^ ions occupying Os and Ts. Table 1 provides the binding-energy positions of the Co^2+^ sub-peaks. The computed percentages of Co^2+^ ions in the two states were 42.08% for state Os and 56.92% for state Ts, respectively, indicating that La-CFO existed as a mixed phase of normal and inverse spinel structures.

Figure 4f shows the detailed XPS spectrum of La-CFO@BC for La 3d. The La 3d spectrum exhibited four peaks. The detection of two distinct XPS peaks, originating from the La 3d_3/2_ and La 3d_5/2_ energy levels, was observed at 851.46 eV and 834.71 eV, respectively. The results align well with previously reported positions of La^3+^ ions [36,37]. In addition, the other two peaks resulted from shake-up satellite features due to interactions between La 4f energy levels and O 2p [38]. These facts all indicated that La in La-CFO@BC exists as La^3+^ [15,39]. Decomposition of La 3d_5/2_ and La 3d_3/2_ peaks yielded four sub-peaks corresponding to La^3+^ ions occupying Os and Ts. Table 1 provided the binding-energy positions of the La^3+^ sub-peaks. The calculated occupancy ratios of La^3+^ in Os and Ts were 53.31% and 46.69%, respectively. In this way, a certain proportion of Fe^3+^, Co^2+^, and La^3+^ form a disordered distribution at the octahedral position, and electrons could quickly transfer between the oxidized states of metal ions, making La-CFO exhibit good conductivity. Within a certain range, as the conductivity increased, the dielectric constant of the material also increased accordingly, which can give an adjustment of the electromagnetic parameters to the material, so that its absorption performance is enhanced.

### 2.2. Magnetic Properties

**Figure 5:**
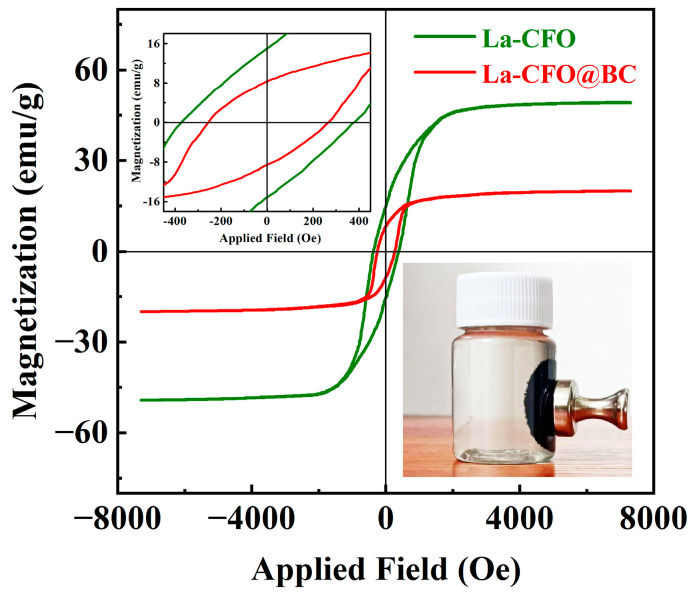
Magnetization curves of La-CFO and La-CFO@BC.

The microwave-absorption performance of a sample is intricately intertwined with its magnetic characteristics. During the absorption of EW, the magnetic components in the material can effectively guide and absorb the electromagnetic waves [40]. Figure 5 presents the magnetic hysteresis loops of La-CFO and La-CFO@BC. Both samples exhibited an S-shaped magnetic hysteresis loop, indicating that they were typical ferromagnets. The coercivity (*H*c) and magnetic saturation (*M*s) values of La-CFO were 378.26 Oe and 49.38 emu/g, respectively. However, La-CFO@BC exhibited significantly reduced values of 264.88 Oe and 19.66 emu/g. This is because there is a non-magnetic component BC in the sample, which results in a relatively small contribution of La-CFO to the magnetic properties per unit mass of the sample. The decrease in coercivity and magnetic saturation of La-CFO@BC allowed microwave energy to be more easily absorbed and transformed into various energy forms, including thermal energy and others. This can improve energy conversion efficiency, which may enable La-CFO@BC to achieve better microwave-absorption performance. This finding highlighted the potential application of La-CFO@BC in materials science and engineering due to its unique chemical and physical properties.

### 2.3. Microwave-Absorption Characteristics

The material’s EW absorption capability is commonly quantified through reflection loss (*R*_L_) [7,41]. According to the theory of reflection transmission, *R*_L_ refers to the loss of energy that is partially reflected back but not absorbed by EW entering a material. Ideally, for efficient absorption, the incident wave should penetrate maximally into the absorber, where it can be effectively dissipated rather than reflected. Consequently, the magnitude of *R*_L_ directly correlates with the absorber’s efficacy in absorbing electromagnetic waves. A larger absolute *R*_L_ value signifies less reflection and a greater proportion of the wave penetrating into the absorber, indicating superior absorption performance [15,42]. The *R*_L_ value could be accurately determined by application of a specified mathematical formulation, grounded in the principles of transmission line theory [43]:

*R*_L_(dB) = 20 log|(*Z*_0_ − *Z*_in_)/(*Z*_0_ + *Z*_in_)|, (1)

where *Z*_0_ represents the fundamental impedance for surrounding vacuum or air, whereas *Z*_in_ signifies the resistive property encountered by an incoming wave at the interface of the microwave-absorption material. Typically, the input impedance (*Z*_in_) of the microwave-absorption material can be derived using the mathematical expression outlined below:

*Z*_in_ = *Z*_0_ (*μ*_r_/*ε*_r_)^1/2^ tanh[*j*(*ε*_r_*μ*_r_) ^1/2^ (2*πdf/c*)] (2)

where the thickness of the absorption layer, denoted by *d*, along with the velocity of EW in a vacuum, *c*, and the frequency of the incoming EW, *f*, play vital roles in determining the performance of the absorber. Additionally, the complex permittivity (*ε*_r_) and complex magnetic permeability (*μ*_r_) of the absorber material are crucial parameters in characterizing its electromagnetic properties. Therefore, for a given incident frequency of electromagnetic waves, optimal impedance-matching and absorption effects could be attained through adjusting the absorbing material’s *d*.

**Figure 6:**
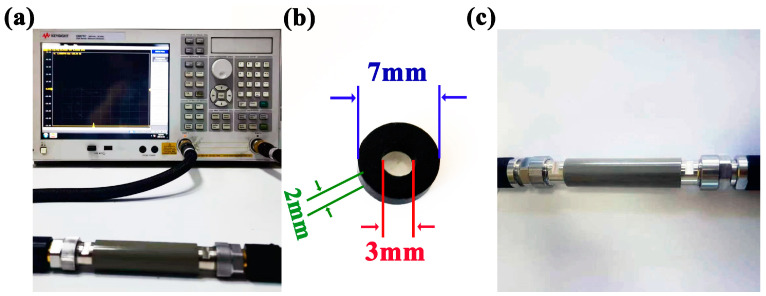
The actual photos of the VAM instrument connection (**a**), the ring-shaped tested sample (**b**), and the coaxial fixture for the tested sample (**c**).

The electromagnetic parameters of the samples are tested by the coaxial method using a vector network analyzer. The paraffin wax and the sample to be tested were mixed in a weight ratio of 7:3. Subsequently, the mixed material was compressed into a circular ring-shaped specimen, possessing an outer diameter measuring 7 mm, an inner diameter of 3 mm, and a thickness precisely set at 2 mm. The electromagnetic parameters *ε*′, *ε*″, *μ*′, and *μ*″ of the material are tested for the sample within the frequency range of 2–18 GHz. Figure 6 shows the actual photos of the VAM instrument connection, the ring-shaped tested sample, and the coaxial fixture for the tested sample.

**Figure 7:**
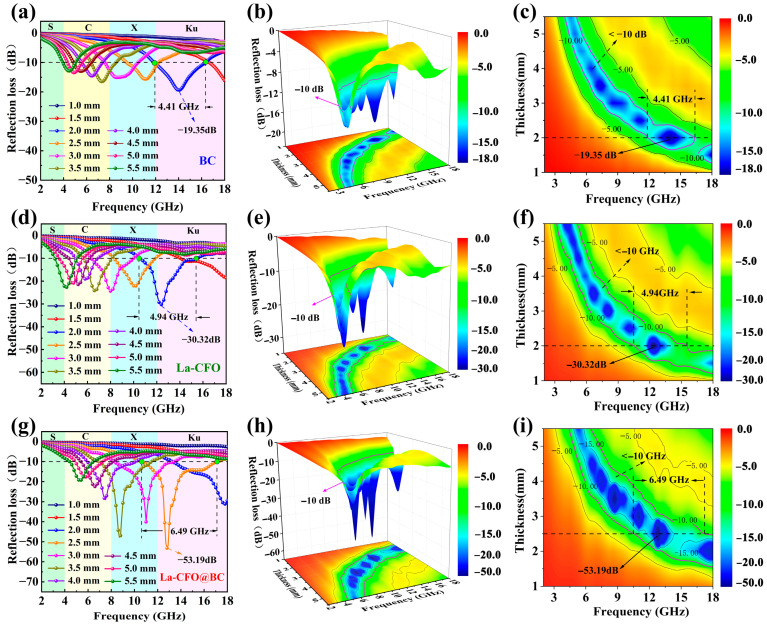
The *R*_L_ curves, *R*_L_ 3D distribution maps and 2D *R*_L_ contour plots of BC (**a**–**c**); La-CFO (**d**–**f**); La-CFO@BC (**g**–**i**).

With the values of *ε*′, *ε*″, *μ*′, and *μ*″ obtained through experiments, the reflection loss *R*_L_ of each sample was derived through Equations (1) and (2). The *R*_L_ curves, along with their corresponding 3D distribution maps and 2D *R*_L_ contour plots were given in Figure 7. Firstly, it could be observed that the *R*_L_ of all samples reached its peak at a certain frequency, regardless of their thickness. And with an increase in thickness, the corresponding minimum peak frequency of the *R*_L_ spectrum gradually decreases. The quarter-wavelength resonance model, described by the given Equation (3), can explain this observed phenomenon [25,44]:

*d*_m_ = *nc*/4*f*_m_ (|*μ*_r_||*ε*_r_|)^1/2^ (3)

In this equation, the parameters *d*_m_ and *f*_m_ designate the respective thickness and frequency of the incident EW, at which the absorbing material attains its *R*_L_ peak. Therefore, *d*_m_ and *f*_m_ exhibit a negative correlation, indicating that as *d*_m_ increases, *f*_m_ decreases.

As depicted in Figure 7, it is evident that the *R*_L_ peak of each sample attains a minimum value *R*_Lmin_. For example, at a frequency of 14.02 GHz, with a 2 mm thickness, sample BC achieved an *R*_Lmin_ of −19.31 dB. This result was similar to that recently reported on absorption effects of biomass carbon (the *R*_Lmin_ of carbonized waste coffee grounds was −20.89 dB at 13.92 GHz, 2.0 mm) [20]. It is noteworthy to mention that the EAB is defined as the frequency range wherein the *R*_L_ value diminishes to less than −10 dB, signifying that a substantial fraction, exceeding 90% of the electromagnetic wave energy is dissipated within that range. A wider EAB indicated a better frequency absorption range and more practical electromagnetic-wave-absorption capabilities. As shown in the Figure 7a–c, sample BC had an EAB of 4.41 GHz, which was also similar to previously reported results (the *R*_Lmin_ of waste coffee grounds was −20.89 dB at 13.92 GHz, 2.0 mm) [20]. According to Figure 7d–f, at a frequency of 14.02 GHz and with a thickness of 2 mm, La-CFO achieved a minimum *R*_L_ of −30.32 dB and possessed an EAB of 4.94 GHz. This was a significant improvement compared to pure CoFe_2_O_4_ particles (at 13.4 GHz and 2.5 mm, *R*_Lmin_ of P-CFO was about −9.91 dB) [15].

As shown in the Figure 7g–i, after loading La-CFO onto BC, the sample La-CFO@BC exhibited a remarkable microwave-absorbing performance, attaining a *R*_Lmin_ value of −53.19 dB at a frequency of 12.80 GHz and a thickness of 2.5 mm, while demonstrating an EAB spanning 6.49 GHz. Table 2 compares some recently reported biomass-carbon-absorbing materials, showing that the synthesized La-CFO@BC magnetic biomass material in this work had certain advantages in terms of absorption amount and EAB. It indicated that La-CFO@BC was a highly promising absorbing material for practical applications.

Overall, the BC provided effective EW absorption in the high frequency range, whereas the magnetic particles played a role in the low and medium frequency ranges. This synergistic effect enabled hybrid materials to effectively absorb electromagnetic waves over a wider frequency range.

**Figure 8:**
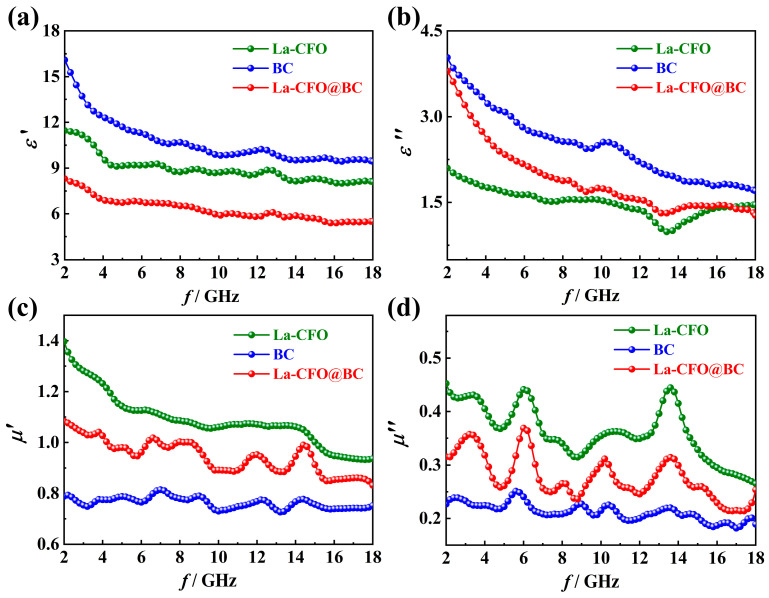
The relationships between frequency and the *ε*′ and *ε*″ (**a**,**b**) as well as *μ*′ and *μ*″ (**c**,**d**) for BC, La-CFO, and La-CFO@BC.

The complex permittivity *ε*_r_ = *ε*′ − *jε*″ and complex permeability *μ*_r_ = *μ*′ − *jμ*″ of a material are closely related to its wave-absorbing performance. The real components ε′ as well as *μ*′ represent the capacity of the material to store the EW energy. Meanwhile, the imaginary components *ε*″ and *μ*″ denote the dissipation capacity of the EW energy, within the material [54,55]. Secondly, when the EW is incident to the material surface, results from the disparity in impedance between the absorber and the surrounding medium, a segment of the EW is reflected to free space but cannot enter the absorbing body [56,57]. The impedance behaviors of the material are dependent on its *ε*_r_ and *μ*_r_. To analyze the absorption mechanism of each sample, Figure 8 gives the frequency-dependent *ε*_r_ and *μ*_r_ of BC, La-CFO and La-CFO@BC using a vector analyzer.

Figure 8a,b illustrates the dielectric parameters *ε*′ and *ε*″ of BC, which were both higher than those of La-CFO. This means that the BC had a stronger dielectric-loss capability compared to that of La-CFO. Moreover, the *ε*′ value together with the *ε*″ value of La-CFO@BC were lower than those of BC alone. This could be ascribed to the introduction of La-CFO with relatively lower *ε*′ and *ε*″ values. It was also noticed that ε″ exhibited a decrease as the frequency rose, a trend that can be traced back to the reduction in space-charge polarization and the consequent decrease in dielectric loss as frequency increases. Additionally, as shown in Figure 8c,d, BC exhibited very low values of *μ*′ and *μ*″ compared to those of La-CFO. This was because BC was a non-magnetic material exhibiting minimal responses to magnetic fields. This observation indicated that BC had a weaker magnetic-loss capability compared to that of La-CFO. The relatively high values of *μ*′ and *μ*″ in La-CFO contributed to the moderate μ′ and μ″ values in the composite material La-CFO@BC. This not only enhanced the magnetic-loss capability of La-CFO@BC but also facilitated the impedance matching.

**Figure 9:**
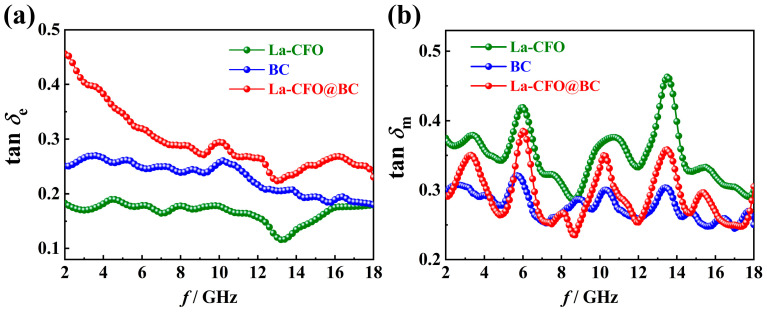
The relationships between frequency and tan*δ*_e_ (**a**) as well as tan*δ*_m_ (**b**) for BC, La-CFO, and La-CFO@BC.

Figure 9a,b depicts the relationships between frequency and the tan*δ*_e_ = *ε*″/*ε*′ as well as tan*δ*_m_ = *μ*″/*μ*′ of BC, La-CFO, and La-CFO@BC, which are usually used to evaluate dielectric and magnetic losses, respectively [44]. As shown in Figure 9a, La-CFO@BC exhibits the highest tan*δ*_e_ value, indicating its high dielectric-loss capability. The observed phenomenon can be ascribed to the thin, layered, and corrugated carbon pore walls within BC, which were serving as conduits that facilitate the formation of a conductive network and were ultimately bolstering the electrical conductivity of La-CFO@BC. Additionally, the pore walls of BC were densely covered with pores of various sizes, ranging from micro to macro, offering an extensive surface area that permitted BC to carry a large amount of La-CFO. After loading, La-CFO@BC possessed a large interface between BC and La-CFO, providing a strong interfacial-polarization capability. In addition, the La-CFO deposited on the surface of BC exhibited both carpet-like and dandelion-shaped morphologies, leading to a significant expansion of the overall surface area of the material. This, in turn, increased the interface between the absorber and its surroundings. As a result, a significant presence of dangling bonds and coordination-unsaturated atoms emerged on the absorber surface, thereby intensifying electronic, ionic, and interfacial-polarization mechanisms within the material. Consequently, La-CFO@BC exhibited a higher electrical polarization rate. Typically, high polarization rates and electrical conductivities result in high dielectric losses [58,59], leading to the higher tan*δ*_e_ = *ε*″/*ε*′ value observed for La-CFO@BC. In addition, reference [60] presents a phenomenological hypothesis regarding the EW absorption capacity of conductive composites, suggesting the existence of a voltage-controlled Fowler-Nordheim tunneling effect. The tunneling phenomenon exerts an influence on the composite material’s effective conductivity via the Drude damping factor, thereby generating alternative current flow routes within the non-conductive resin matrix. Consequently, these extra current paths effectively transform the resin into a virtually conductive medium, thereby altering the effective dielectric constant of the two-phase composite material. In our composite material, La-CFO@BC, numerous gaps exist during the combination of BC and La-CFO. It is possible that a tunneling effect occurs within these gaps, influencing the composite’s effective conductivity through the Drude damping factor and modifying its effective dielectric constant. This, in turn, has the potential to enhance the material’s absorption of electromagnetic wave energy.

It can also be observed in Figure 9b that La-CFO exhibited the highest tan*δ*_m_ value. This phenomenon stemmed from the intrinsic properties of CFO ferrite materials, including significant eddy current loss, magnetic aftereffect loss, magnetic hysteresis loss, domain wall resonance loss, and natural resonance-loss capabilities. Moreover, the incorporation of the element La increased the coercivity and magnetic anisotropy field of the CFO ferrite crystal, thereby enhancing its magnetic hysteresis loss. Furthermore, compared to Fe and Co ions, the larger radius of La ions exerted a pinning effect on domain wall motion, enhancing the natural resonance absorption and the domain wall resonance absorption of CFO ferrite. Additionally, the rare-earth element La had a numerous atomic energy level. When it was doped into the ferrite crystal, it increased the density of material energy levels and also enhanced the opportunity for energy level transitions. This increased energy transfer between ions and improved the multi-band energy absorption capability of the absorbing material. Compared to BC and La-CFO, the sample La-CFO@BC exhibited an intermediate tan*δ*_m_ value while maintaining a similar trend to La-CFO. It indicated that La-CFO@BC possessed a certain magnetic loss capability and exhibited magnetic-loss patterns similar to those of La-CFO. By comparing the tan*δ*_m_ plot of La-CFO@BC (Figure 9b) with its reflection loss plot (Figure 7g), it could be observed that the tan*δ*_m_ of the sample peaked at a frequency of 13.20 GHz, which was very close to the frequency corresponding to its minimum loss peak at 12.80 GHz. Therefore, it could be concluded that the improvement in microwave-absorption capability of La-CFO@BC was predominantly attributed to the augmented magnetic loss.

**Figure 10:**
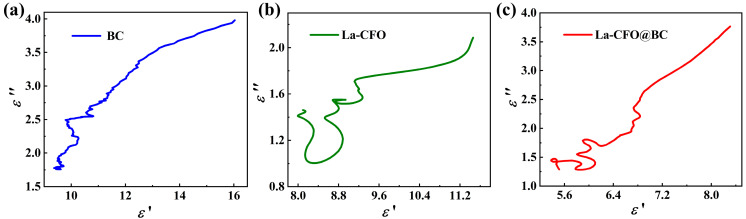
Cole–Cole curves of (**a**) BC, (**b**) La-CFO, and (**c**) La-CFO@BC.

In addition, the Debye relaxation model provides the basis for Equation (4) [61],

(4) $${\left[\varepsilon^{\prime }-\left(\varepsilon_{\infty }+\varepsilon_{s}\right)/2\right]}^{2}+{\left(\varepsilon \prime \prime \right)}^{2}={\left[\left({\varepsilon_{\infty }-\varepsilon }_{s}\right)/2\right]}^{2}$$

where *ε*_s_ represents the stationary dielectric permittivity, whereas *ε*_∞_ denotes the limiting relative dielectric constant at high frequencies. Figure 10 presents the Cole–Cole plot of *ε*″ as a function of *ε*′. Typically, the presence of a semicircle on the Cole–Cole plot signifies the occurrence of a polarization-relaxation mechanism, whereas the emergence of multiple semicircles denotes the concurrent occurrence of numerous polarization-relaxation processes. As can be seen from Figure 10, there are numerous Cole–Cole semicircles in all three samples, with La-CFO and La-CFO@BC showing more semicircles, indicating that multiple polarization-relaxation processes existed in all samples, with La-CFO and La-CFO@BC having more polarization-relaxation processes. Additionally, it could be observed that the Cole–Cole curves of BC and La-CFO@BC exhibited a linear long tail, indicating the presence of strong conductive losses [19,62]. This phenomenon could potentially originate from the conductive losses associated with the conductive network established by BC.

According to the theory of transmission lines, when incident EW in free space reaches the absorbing body, they may be partially reflected due to impedance mismatch. To facilitate the efficient absorption of EW energy by the absorbing body, it is imperative to minimize the reflection of EW and maximize its penetration into the interior of the absorbing material. This requires the establishment of an optimal impedance match between the absorber and the surrounding free space. The effect of impedance matching is usually evaluated using the parameter *Z* (Equal to 1 in ideal matching), which can be obtained by converting Equation (5) from Equation (2).

(5) $$Z=\left|\frac{Z_{\mathrm{in}}}{Z_{0}}\right|={\left(\frac{\mu_{r}}{\varepsilon_{r}}\right)}^{1/2}\;\tanh[j\left(\varepsilon_{r}\mu_{r}\right){\;}^{1/2}\;(\frac{2\pi df}{c})]$$

**Figure 11:**
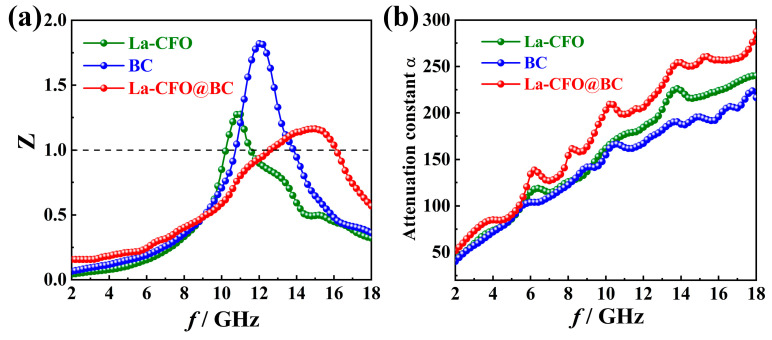
*Z* (**a**) and α (**b**) in relation to frequency for BC, La-CFO, and La-CFO@BC.

The thicknesses of BC, La-CFO, and La-CFO@BC were fixed as 2.5 mm, so that their *Z* values were calculated based on Equation (5). Figure 11a shows the *Z*-*f* curve of each sample. One can observe that, compared to the other two samples, the *Z* value of La-CFO@BC was closer to 1, and the spectral band corresponding to the *Z* value close to 1 was broader. Therefore, EM waves more easily penetrated into the interior of La-CFO@BC absorbers than into the other materials. This result indicated that the EM parameters of the hybrids could be adjusted by rational composite mixing of BC with La-CFO to achieve superior impedance-matching characteristics [63]. Another significant characteristic parameter that characterizes the performance of the absorber is its ability to absorb electromagnetic waves internally, denoted as α. This parameter α can be expressed mathematically using Equation (6) [64]:

(6) $$\alpha =\left(\sqrt{2}\pi f/c\right)\times {\left[\left(\mu^{\prime \prime }\varepsilon^{\prime \prime }-\mu^{\prime }\varepsilon^{\prime }\right)+\sqrt{{{\left({\varepsilon^{\prime }\mu }^{\prime \prime }+\mu^{\prime }\varepsilon^{\prime \prime }\right)}^{2}+({{\varepsilon^{\prime }\mu^{\prime }-\varepsilon }^{\prime \prime }\mu }^{\prime \prime })}^{2}}\right]}^{1/2}$$

Referring to Figure 11b, the frequency-dependent α-value curves of the three samples, which were computed based on electromagnetic parameters, are depicted. It is observable that the α-value of La-CFO@BC was generally higher than that of BC and La-CFO. Based on the previous analysis, the high α-value of La-CFO@BC could be attributed to the strong interface polarization between BC and La-CFO, in conjunction with the collaborative actions of dipole polarization, electron transport, and magnetic resonance, ultimately enhancing the EW absorption capacity of La-CFO@BC.

**Figure 12:**
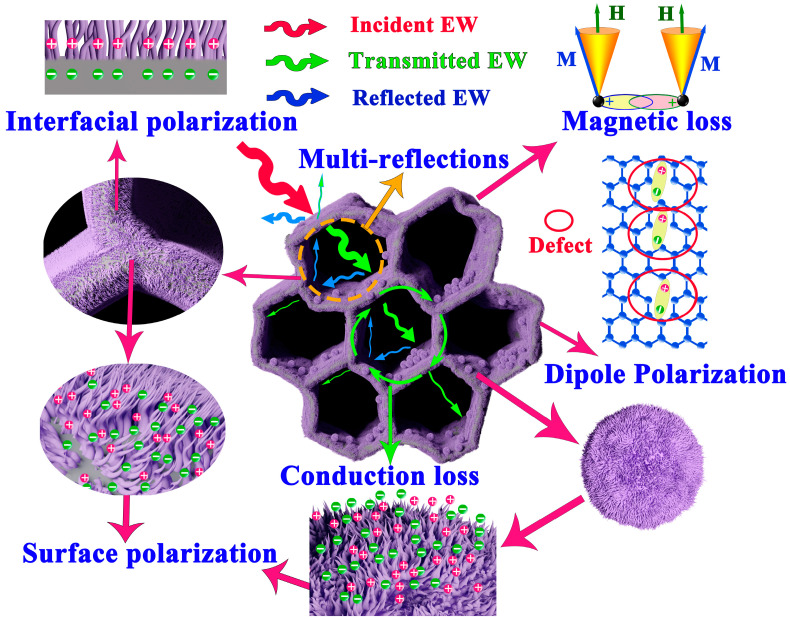
The schematic for the potential EW-loss process of La-CFO@BC.

Based on the above experimental data and on discussions considering the unique morphology of La-CFO@BC, the potential absorption process of the La-CFO@BC was illustrated in Figure 12. The thin, corrugated carbon pore walls of La-CFO@BC form a 3D=-interconnected conductive network. The presence of numerous micropores and mesopores on these pore walls, along with defects introduced by the loading of La-CFO, provide appropriate resistance and capacitance to the conductive network. Under the influence of incident electromagnetic waves, this 3D conductive network can act as a resistive-inductive–capacitive coupling circuit, generating time-varying electromagnetic induction currents. These complex, long-distance induction currents decay as they propagate through the 3D network, converting electromagnetic energy into thermal energy, thus contributing to electrical-conduction loss. Moreover, La-CFO@BC exhibits a diverse porosity distribution, allowing EW to undergo numerous reflections in the material’s micropores, mesopores, and macropore channels, leading to multiple reflection phenomena. This increases the depth and area of electromagnetic wave propagation within the absorbing material, enabling the absorption of as much electromagnetic energy as possible. Furthermore, the integration of BC and La-CFO enhances the composite’s overall impedance-matching profile, balancing its magnetic-loss capacity and dielectric-loss capacity. From another perspective, the rich polarization enhances absorber’s dielectric-loss ability. Firstly, the formation of numerous defects during the loading of La-CFO onto BC promotes the generation of internal dipoles, resulting in rich dipole polarization. Secondly, the numerous contact interfaces between BC and La-CFO provide abundant interfacial polarization for the material. Finally, the carpet-like and dandelion-like surface textures of La-CFO deposited on the material provide significant surface polarization. Therefore, the doping with the rare-earth element La enhances the magnetic-loss capacity of the composite material, broadening its effective absorption frequency bandwidth. The unique carpet-like and dandelion-like morphologies of La-CFO also contribute to enhancing the magnetic-loss capacity of the material, thus playing important roles in absorbing magnetic field energy. Based on these characteristics, La-CFO@BC exhibits excellent absorption properties.

## 3. Materials and Methods

### 3.1. Preparation of the Composites

Firstly, the treated corn stalks were heated at a rate of 10 °C per minute in a vacuum tube furnace under N^2^ atmosphere to the constant temperature of 450 °C, and then calcined for 3 h. Once the temperature had gradually and naturally subsided to ambient room temperature, the carbonized corn stalks (BC) were taken out. The BC was then activated and treated. Subsequently, 1.5 g of the treated BC, 1.3 g of NaAc (Phygene, Fuzhou, China), and 1 g of polyethylene glycol (Aladdin, Shanghai, China) were added into 70 mL of ethylene glycol (EG, Aladdin, Shanghai, China) and stirred for 10 min. Then, according to the stoichiometric ratio of CoLa_0.2_Fe_1.8_O_4_, a total amount of 2.0 g of Fe(NO_3_)_3_·9H_2_O, La(NO_3_)_3_·6H_2_Oand Co(NO_3_)_3_·6H_2_O (Aladdin, Shanghai, China) were introduced to the solution. The mixed solution was magnetically stirred for another 10 min and sonicated for 30 min, then poured into a stainless-steel autoclave liner, which was kept at 140 °C for 24 h, subsequently reaching ambient temperature naturally. The mixed solution underwent filtration to yield a black substance; after undergoing numerous ethanol rinses, the material was then dehydrated in an oven kept at a temperature of 80 °C, ultimately resulting in La-CFO@BC (the weight ratio of the CoLa_0.2_Fe_1.8_O_4_ in the La-CFO@BC was 20%). In addition, for comparative experiments, a similar procedure was employed to synthesize pure CoLa_0.2_Fe_1.8_O_4_ without loading on BC, which was labeled as La-CFO.

### 3.2. Characterization

The synthesized samples were subjected to various analytical techniques to measure their physical and chemical properties. X-ray diffraction methodology (XRD, PANalytical Empyrean, Almelo, The Netherlands) was applied to determine the phase structure of the product, with a scan range set from 10° to 80° in 2*θ*. Raman spectrometer spectroscopy (RAM, Thermo Fisher, Waltham, MA, USA) was used to analyze the molecular structure information of BC. To examine the physical appearance and microscopic characteristics of the product outcomes, scanning electron microscopy (SEM, Thermo Scientific Apreo 2C, Waltham, MA, USA) as well as transmission electron microscopy (TEM, FEI Tecnai F20, Hillsboro, OR, USA) were utilized. The chemical configurations of the product were examined through the utilization of X-ray photoelectron spectroscopy (XPS, Thermo Fisher Escalab 250 xi, Waltham, MA, USA). The XPS spectra were calibrated with reference to the C 1s signal at 284.6 eV. The N_2_ adsorption–desorption isotherm analysis was conducted utilizing a specialized surface area and pore-size analyzer at −196 °C (BET, Micromeritics Asap 2020, Norcross, GA, USA). An assessment of the magnetic characteristics of the specimens was conducted using a physical property measurement system (PPMS, Quantum Design Inc., San Diego, CA, USA). Using a vector network analyzer (VNA, Agilent E5071c, Santa Clara, CA, USA), the electromagnetic parameters of the sample were measured utilizing the coaxial method within the frequency range of 2–18 GHz. For the purpose of conducting the tests, the paraffin wax and the sample were blended together in a ratio of 7 parts wax to 3 parts sample, by weight. The blended mixture was then compressed into circular rings, featuring an internal diameter of 3 mm, an external diameter of 7 mm, and a height of 2 mm.

## 4. Conclusions

Biomass carbon (BC) was prepared from corn stalks, and rare-earth-doped spinel ferrite La-CFO was directly loaded on BC via a hydrothermal method, resulting in the successful synthesis of a low-density composite for microwave absorption, La-CFO@BC. The composite not only retained the characteristics of low density and abundant porosity of typical biomass-carbon materials but also exhibited a unique morphology. Specifically, La-CFO covering the BC surface displayed a carpet-like structure with dandelion-shaped La-CFO particles embedded within it. The loading of La-CFO modulated the electromagnetic parameters of the composite, addressing the weakness of poor magnetic loss in pure BC materials. As a result, La-CFO@BC exhibited superior impedance-matching characteristics and displayed outstanding microwave-absorption capability. Under the conditions of an incident EW frequency of 12.8 GHz, the *R*_Lmin_ value of La-CFO@BC reached −53.2 dB at a thickness of 2.5 mm, with an EAB of 6.4 GHz. By varying the thickness of the composite between 1.0 and 5.5 mm, the effective absorption bandwidth of La-CFO@BC could be extended to 13.8 GHz, encompassing the entire X-band, the entire Ku-band, and most of the C-band electromagnetic waves. The findings of this work may thus provide a substantial advancement in the field, offering a promising alternative to conventional microwave absorbers.

## Figures and Tables

**Table 1:** The XPS sub-peak positions of Fe 2p, Co 2p, and La 3d for the La-CFO@BC.

	Energy Position (eV)						
Spectrum		Fe 2 p_1/2_	Fe 2p_3/2_	Co 2p_1/2_	Co 2p_3/2_	La 3d_3/2_	La 3d_5/2_
Assignment	Os	724.81	710.85	796.28	780.33	850.86	834.11
	Ts	726.20	712.21	798.16	782.21	852.12	835.37

**Table 2:** Comparative analysis of microwave-absorbing performance for different biomass-carbon materials.

Feedstock	Samples	*R*_Lmin_ Value (dB)	EAB (GHz)	Thickness at EAB (mm)	Ref.
Soybean dregs	FK-SDC	−18.50	4.80	3.50	[45]
Shaddock peel	G-800	−29.50	5.80	1.70	[46]
Kapokfibers	CMT-900	−30.75	6.78	2.06	[19]
Cotton fibers	PCMT/Co	−36.8	6.7	1.4	[47]
Wheat flour	PCP@Ni-chain	−38.42	5.2	2.0	[42]
Alginate	Ni/Ni_3_ZnC_0.7_/C-0.1	−40.2	5.4	2.0	[41]
Rice husk	BHPC	−47.46	3.4	2.8	[48]
Bamboo	GC-8	−51.00	4.2	1.66	[49]
Shaddock peels	Fe_3_O_4_/PCS-2	−50.3	4.1	1.6	[50]
Humins	CS@C-700	−51.4	5.2	1.84	[51]
Coffee grounds	WCG-20–750	−52.86	6.40	3.0	[20]
Corn stalks	La-CFO@BC	−53.19	6.49	2.5	This work
Chestnut needles	VO_2_–50	−54.0	2.5	2.87	[52]
Pine needles	PNC	−56.30	3.44	1.4	[53]

## Data Availability

Data of the compounds are available from the authors.
